# Characterization, histopathology and immunogenicity of the lumpy skin disease virus isolated during 2019–20 in Bangladesh

**DOI:** 10.3389/fmicb.2024.1324243

**Published:** 2024-04-25

**Authors:** Mohammad Asir Uddin, Muhammad Tofazzal Hossain, A. K. M. Anisur Rahman, Mahbubul Pratik Siddique, Md. Abdul Kafi, Md. Golbar Hossain, Sourav Chakraborty, Mohummad Muklesur Rahman, A. K. M. Khasruzzaman, Michael P. Ward, Md. Alimul Islam

**Affiliations:** ^1^Department of Microbiology and Hygiene, Bangladesh Agricultural University, Mymensingh, Bangladesh; ^2^Department of Medicine, Bangladesh Agricultural University, Mymensingh, Bangladesh; ^3^Department of Veterinary and Animal Sciences, University of Rajshahi, Rajshahi, Bangladesh; ^4^Sydney School of Veterinary Science, The University of Sydney, Camden, NSW, Australia

**Keywords:** embryo inoculation, P32 gene, iELISA, phylogenetic analysis, LSDV, iiPCR, PCR

## Abstract

**Introduction:**

Lumpy skin disease (LSD) is a highly contagious vector-borne viral disease of cattle. LSD has emerged in Bangladesh in 2019, causing significant economic losses due to its high morbidity and mortality. This research was designed to isolate, identify, and assess the immunogenicity of LSD virus (LSDV) using nodular tissue samples obtained from affected cattle during the 2019–20 outbreak across nine districts of Bangladesh.

**Methods:**

To determine the presence of LSDV in nodular tissues, we initially used iiPCR and PCR, followed by histopathological examination. 151 were positive via iiPCR and PCR among the 180 collected samples. The PCR positive 151 samples were then inoculated into 10-day-old embryonated chicken eggs via the CAM route to isolate LSDV, confirmed through PCR. Subsequently, partial sequencing and phylogenetic analysis of the P32 gene were performed to determine the origin of the circulating LSDV strain. The immunogenicity of selected LSDV strains was assessed through an ELISA test.

**Results:**

The PCR results revealed a distinct positive band at 192 bp in both the nodular tissue samples and the LSDV isolated from chicken embryo inoculations. Microscopic analysis of the nodular lesions revealed thickening of the epidermis, ballooning degeneration of keratinocytes, and proliferation of follicular epithelia. Additionally, mononuclear infiltration was observed at the demarcation line between infected and healthy tissue, with necrosis of muscular tissues beneath the epidermis. The LSDV isolate from Bangladesh exhibited a close genetic relationship with LSDV strains isolated from neighboring and other regional countries including India, Myanmar, and Mongolia. This observation strongly suggests the possibility of a transboundary spread of the LSD outbreak in Bangladesh during 2019–2020. The results of the immunogenicity test showed that the serum antibody titer remained at a protective level for up to 18 months following secondary immunization with inactivated LSDV antigen. This finding suggests that the inactivated LSDV antigen could be a potential vaccine candidate to protect cattle in Bangladesh against LSDV.

**Conclusion:**

In conclusion, our research successfully isolated, identified, and characterized LSDV in cattle nodular tissues from the 2019–20 outbreak in Bangladesh. Furthermore, it provided insights into the probable origin of the circulating strain and investigated a potential vaccine candidate to protect cattle in the region from LSDV.

## Introduction

Lumpy Skin Disease (LSD) is an important transboundary viral disease of livestock with substantial economic implications. It is caused by the Lumpy Skin Disease Virus (LSDV), which belongs to the genus Capripoxvirus, subfamily Chordopoxvirniae, and family Poxviridae (Uddin et al., 2022). LSD is highly host-specific, primarily affecting cattle (both *Bos indicus* and *B. taurus*), although it can also infect water buffalo (*Bubalus bubalis*) (Yousefi et al., 2017;World Organization for Animal Health, 2023). Notably, LSDV does not infect or spread between sheep and goats (World Organization for Animal Health, 2023). The clinical presentation of LSD varies widely, ranging from asymptomatic illness to fatal outcomes (Carn and Kitching, 1995). Common clinical features of LSD include high fever, loss of appetite, generalized skin nodules, enlarged lymph nodes, sterility, skin erythema, and abortion (Uddin et al., 2022). The severity of the disease can vary depending on factors such as the LSDV strain, cattle breed, sex, age, with lower mortality typically below 2% and variable morbidity that can reach up to 100% (Sprygin et al., 2018; Badhy et al., 2021; Wilhelm and Ward, 2023). In southeast Asia during 2020–21, average estimated morbidity and mortality of 21 and 2.68%, respectively, were estimated (Wilhelm and Ward, 2023).

Originally, LSD was an endemic disease in numerous African countries, and it subsequently expanded from sub-Saharan Africa to the Middle East. It then continued to spread into Europe and Asia (Milovanović et al., 2019). The first reports of LSD outbreaks in Bangladesh and India came in July and August 2019, respectively. These outbreaks further spread to nearly all Central, South, and Southeast Asian countries (Parvin et al., 2022; Uddin et al., 2022). By 2019–20, LSD had reached virtually every district in Bangladesh, emerging as an important disease (Haque and Gofur, 2020; Sarkar et al., 2020; Badhy et al., 2021; Hasib et al., 2021; Khalil et al., 2021; Chouhan et al., 2022; Uddin et al., 2022). This widespread outbreak inflicted substantial financial losses on the livestock industry due to hide damage and a decrease in body mass. LSD has significant economic impact on the livestock industry due to its rapid spread in recent years. Its continued spread poses a global threat, evident in regions such as Africa, the Middle East, and Asia, with Western Europe and Australia now facing imminent risk. Due to its fast-expanding nature the potential for extensive losses in livestock is unavoidable (Akther et al., 2023).

Several methods to detect the LSDV genome are available worldwide, including PCR, real-time PCR, and HRM-based techniques. In Bangladesh, PCR (Giasuddin et al., 2019; Badhy et al., 2021) and real-time PCR (Parvin et al., 2022) have been utilized for the detection and phylogenetic analysis of LSDV. Parvin et al. (2022) also conducted gross and microscopic pathology examinations of skin nodules in affected cattle. However, there have been no studies on LSDV isolation in chicken embryos or cell culture in Bangladesh. A recent technique, insulated isothermal PCR (iiPCR), has been used for the detection of various veterinary viruses (Lung et al., 2016; Zhang et al., 2019; Song et al., 2022). This method induces spontaneous fluid convection in a capillary tube, ensuring efficient denaturation, annealing, and extension of the PCR process (Tsai et al., 2012). iiPCR shows sensitivity equivalent to real-time PCR and provides clear positive or negative results on the device screen after data processing. In this study we isolated LSDV using chicken embryos after initial screening of field samples using iiPCR and PCR. We also conducted pathology and phylogenetic analyses of the embryo-adapted isolate and assessed its antigenicity.

**Figure 1:**
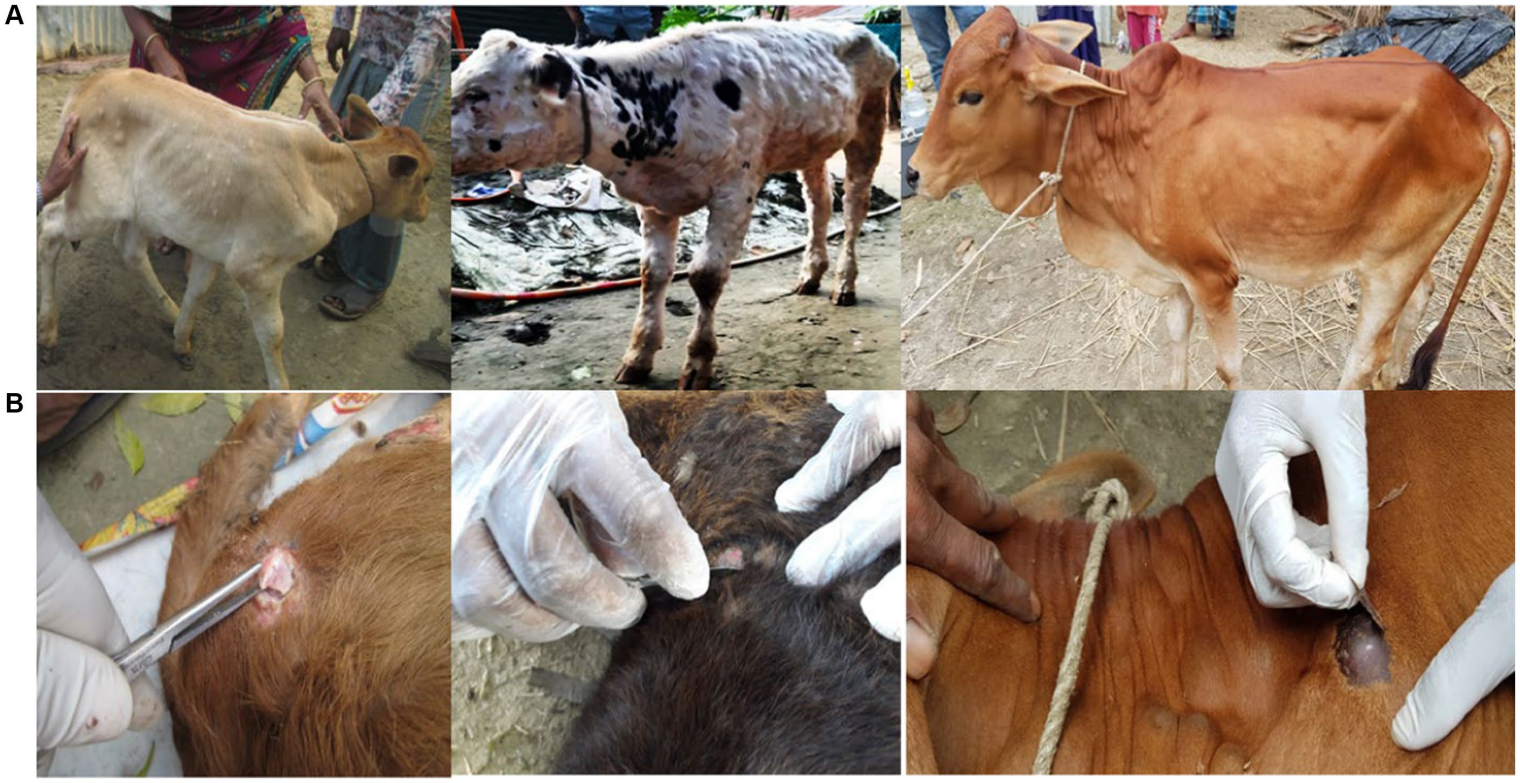
LSD affected cattle **(A)** and collection of skin nodular tissues from LSD affected cattle **(B)**.

## Materials and methods

### Sample collection

**Figure 2:**
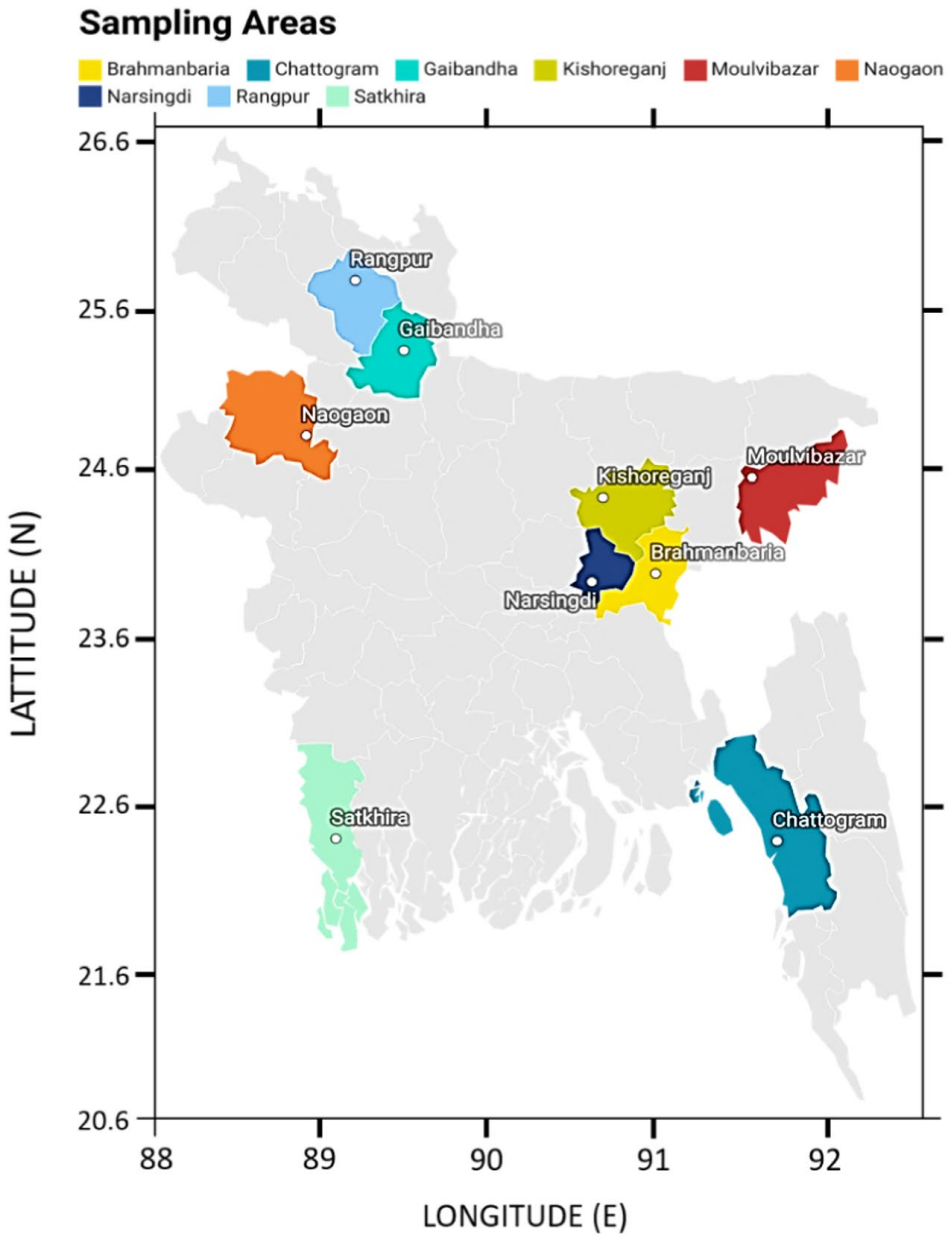
Map of Bangladesh showing the sampling areas, Brahmanbaria (24.1585° N, 89.4481° E), Chattogram (22.3350° N, 91.8325° E), Gaibandha (25.3290° N, 89.5415° E), Kishoreganj (24.4331° N, 90.7866° E), Moulvibazar (24.4808° N, 91.7644° E), Naogaon (24.8000° N, 88.9333° E), Narsingdi (23.9167 ° N, 90.7167° E), Rangpur (25.7500° N, 89.2444° E), and Satkhira (22.7185° N, 89.0705° E).

Clinical signs of the LSD suspected cattle were skin nodules, fever, depression with anorexia, weight loss, reduced milk yield and peripheral lymphadenopathy. A total of 180 skin nodular tissues were collected from cattle suspected to be affected with LSD, all of which were under 2 years of age (see Figure 1). These cattle were located in nine different districts in Bangladesh: Brahmanbaria, Chattogram, Gaibandha, Kishoreganj, Moulvibazar, Naogaon, Narsingdi, Rangpur, and Satkhira. These districts were chosen because LSD outbreaks occurred there during the study period. The sample collection was conducted within 4–30 days of infection during the 2019–20 outbreaks (Table 1; Figure 2). All samples were collected using aseptic techniques, transported with ice carriers and then stored at −80°C for the isolation of LSDV. In addition, 18 skin nodule biopsies, with two samples collected from each of the nine districts, were obtained from the suspected cases. These biopsies were placed in plastic containers containing 10% neutral buffered formalin for subsequent histopathological analysis (Slaoui and Fiette, 2011).

**Table 1:** The distribution of nodular tissue samples that tested positive for iiPCR and PCR across different geographic areas.

	Districts									Total
	Brahmanbaria	Chattogram	Gaibandha	Kishoreganj	Moulvibazar	Naogaon	Narsingdi	Rangpur	Satkhira	
Samples tested	30	14	5	55	28	2	32	10	4	180
iiPCR and PCR positive	24	11	4	47	23	2	27	9	4	151
Detection frequency (%)	80.0	78.6	80.0	85.5	82.1	100	84.4	90	100	83.88

### Sample preparation

The nodular tissue samples were homogenized with sea sand using mortar and pestle, and subsequently 20% suspensions were prepared by adding sterile phosphate buffered saline. The suspension was then centrifuged at 3000 rpm for 10 min maintaining a temperature of 4°C (Zinnah et al., 2010). After centrifugation, the supernatant was carefully collected and stored at -20°C. A portion of this supernatant was used for the extraction of LSD viral DNA, while the remainder was treated with antibiotics (penicillin 10,000 IU/mL + streptomycin 10,000 μg/mL) for the propagation of LSD virus in chicken embryo.

### LSD viral DNA extraction from nodular tissue, chicken embryo and CAM for screening and detection of LSDV by iiPCR and PCR

The genomic viral DNA was extracted from the collected nodular tissues, chicken embryo and chorioallantoic membrane (CAM) using the DNeasy Blood & Tissue kit (Qiagen, Germany) following the manufacturer’s instructions for molecular studies. The elution of DNA was carried out using 70 μL of elution buffer, and the extracted DNA was stored at -20°C for subsequent use.

### Insulated isothermal PCR (iiPCR)

To detect LSDV nucleic acids, we conducted iiPCR using the PockitTM LSDV reagent set (GeneReach Biotechnology Corp., Taiwan) (Chang et al., 2012; Tsai et al., 2012). In brief, we combined 50 μL of premix buffer with 5 μL of the extracted genomic DNA in an R-tube. After a brief centrifugation using the cubee™ mini-centrifuge (GeneReach Biotechnology Corp., Taiwan), the R-Tube was loaded onto the POCKITTM nucleic acid analyzer. The default program included a step at 50°C for 10 min followed by 95°C for 48 min. Fluorescent signals were collected, and signal-to-noise (S/N) ratios were calculated by dividing signals collected after iiPCR by those collected before iiPCR. The results were then automatically displayed on the screen as “Positive” (+), “Negative” (−), or “Unknown” (?) based on the default S/N thresholds.

### PCR

All the nodular tissue from LSD suspected cattle were initially screened for the presence of LSDV genome using the forward primer 5′-TCCGAGCTCTTTCCTGATTTTTCTTACTAT-3′ and reverse primer 5′-TATGGTACCTAAATTATATACGTAAATAAC-3′. These primers amplify a 192-bp fragment of the P32 envelope protein gene (LSDV074). A total volume of 25 μL PCR mixture was prepared consisting of 12.5 μL of master mix (Bio-Rad, United States), 1 μL (10 pmol) of each forward and reverse primers, 5.5 μL of nuclease-free water, and 5 μL of template DNA. The PCR assays were carried out in a thermal cycler (T-1000, Bio-Rad, USA) with the following cycling conditions: an initial cycle at 94°C for 5 min, followed by 35 cycles at 94°C for 1 min, 50°C for 30 s, and 72°C for 1 min. This was then followed by a final cycle at 72°C for 5 min. The PCR products were subsequently analyzed by electrophoresis on a 1.5% agarose gel-containing ethidium bromide (1 μg/mL) in 1x TAE buffer.

### Histopathological examination

The nodular skin tissues were fixed in 10% neutral buffered formalin, subjected to dehydration using increasing concentrations of alcohol, cleaned with xylene, and then embedded in paraffin. The paraffin embedded tissues were subsequently sectioned at a thickness of 5 μm and stained with hematoxylin and eosin.

### Chicken embryo inoculation

A total of 26 PCR-positive nodular tissue samples, three samples from eight districts and two from another district, were inoculated into 10-day-old embryonated chicken eggs using the chorioallantoic membrane route (Akter et al., 2004; Hossain et al., 2005; Ahamed et al., 2015). In cases in which pock lesions were observed on the chorioallantoic membrane, allantoic fluid, the embryo, and the chorioallantoic membrane were collected and processed. Subsequently, PCR was again performed to confirm the presence of LSDV in the collected samples from dead embryos.

### Amplification and sequencing of the RPO30 gene

For one isolate from Brahmanbaria district (embryo-adapted), PCR was carried out using a specific pair of primers: CpRPO30F (forward): 5’-CAGCTGTTTGTTTACATTTGATTTTT-3′ and CpRPO30R (reverse): 5’-TCGTATAGAAACAAGCCTTTAATAGA-3′ (Gelaye et al., 2015; Badhy et al., 2021). The PCR reaction was conducted in a total volume of 25 μL, consisting of 500 nM forward primer, 500 nM reverse primer, 0.2 mM dNTPs, 1 PCR buffer (Qiagen), 2.5 U of Taq Polymerase (Qiagen), and 5 μL of template DNA. The PCR involved initial denaturation at 95°C for 4 min, followed by 40 cycles at 95°C for 30 s, 56°C for 30 s, and 72°C for 45 s, concluding with a final extension at 72°C for 7 min. The PCR products were separated by electrophoresis on a 1.5% agarose gel at 100 V for 60 min and visualized using a Gel Documentation System (Bio-Rad, United States). The PCR amplicons were purified using the Wizard SV Gel and PCR clean-up system kit (Promega) according to the manufacturer’s instructions. LGC Genomics (Germany) performed the sequencing of the purified PCR amplicons. Vector NTI 11.5 software (Invitrogen, USA) was used for sequencing data analysis and assembly.

### Phylogenetic analysis

The nucleotide sequences were aligned using the Muscle algorithm, and the codon option was applied using MEGA7 (Kumar et al., 2016). Subsequently, TreeAnnotator was utilized to generate the Maximum Clade Credibility (MCC) tree, with a 3% burn-in discarded. The resulting tree, along with its associated meta-data, was visualized using the ggtree package in R (Yu et al., 2017).

### Immunogenicity test of the selected LSD virus antigen

The LSDV isolate was propagated in chicken embryos in large quantities. The allantoic fluid, which contained LSDV, was collected and subjected to centrifugation, filtration, and inactivation using 0.3% formalin. The inactivated LSDV was then mixed with montanide MS 130 (SEPPIC, China) for inoculation into experimental cattle. The initial immunization was inoculated into ten cattle at a rate of 1 mL subcutaneously. After 28 days from the first immunization, a booster dose was administered using the same 1 mL dose. In addition, ten cattle were vaccinated once with goat pox vaccine, while another five cattle were used as control subjects. Blood samples were collected for sera at intervals of 1, 2, 9, and 18 months after immunization. The detection of antibodies through ELISA was conducted using the ID Screen® Capripox double antigen Multi-species ELISA kit from ID.Vet® (Grabels, France), following the manufacturer’s instructions.

## Results

### Detection of LSDV in field samples

**Figure 3:**
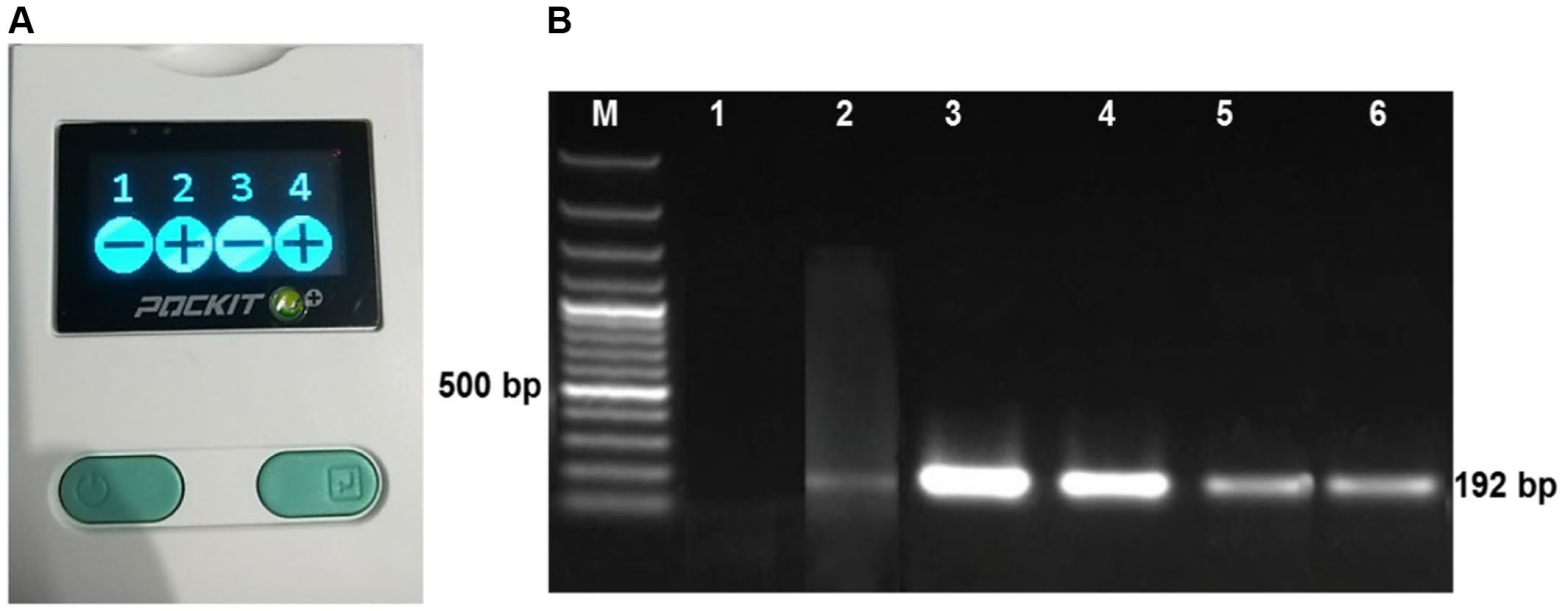
LSDV screening from field sample by iiPCR **(A)** and PCR **(B)**. 1 & 3: LSD Negative sample, 2 & 4: LSD positive samples **(A)** and M: 100 bp DNA marker, Lane 1: negative control, Lane 2: positive control, Lane 3 to 6: the fresh nodules/lumps showed positive band at 192 bp **(B)**.

Of the 180 nodular tissue samples (Table 1), 151 were confirmed positive for LSDV via both iiPCR (Figure 3A) and PCR (Figure 3B). A positive band at 192 bp was consistently observed for all positive cases, indicating the presence of LSDV (Figure 3B). The overall isolation frequency was 83.9%.

### Histopathological analysis

**Figure 4:**
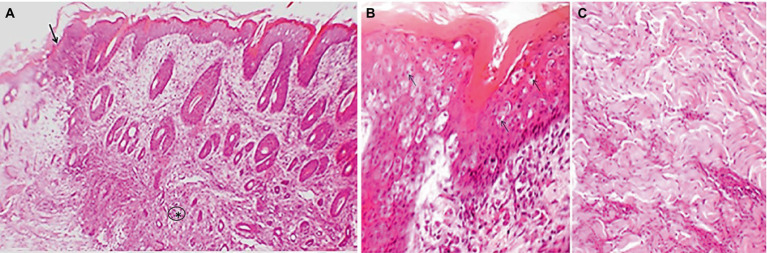
Representative image showing microscopic lesions of lumpy skin disease in cattle. The epidermis is thickened with ballooning degeneration of keratinocytes, proliferation of follicular epithelia and thickening and degeneration of subcutaneous muscular tissue (asterisk), the dermis is infiltrated with foci of inflammatory cells (arrow) mainly involving mononuclear cells at the junction between healthy and nodular tissue **(A)**. The degenerated cells of the epidermis often contain intracytoplasmic inclusion body (**B**, arrows). The muscular tissue in the dermis is degenerated **(C)**. Hematoxylin and eosin stain.

Grossly, nodular skin lesions were up to 2–5 cm in diameter and were elevated above the skin surface. Microscopically, the nodular lesions consisted of epidermal thickening, ballooning degeneration of the keratinocytes, and proliferation of follicular epithelia (Figure 4). The degenerated keratinocytes often contained intracytoplasmic eosinophilic inclusion bodies. Additionally, there were mononuclear inflammatory infiltrates observed at the demarcation line between the lesion and the adjacent healthy tissue. Moreover, the muscular tissues situated beneath the epidermis showed signs of necrosis.

### Isolation of LSDV using chicken embryo and reconfirmation by PCR

**Figure 5:**
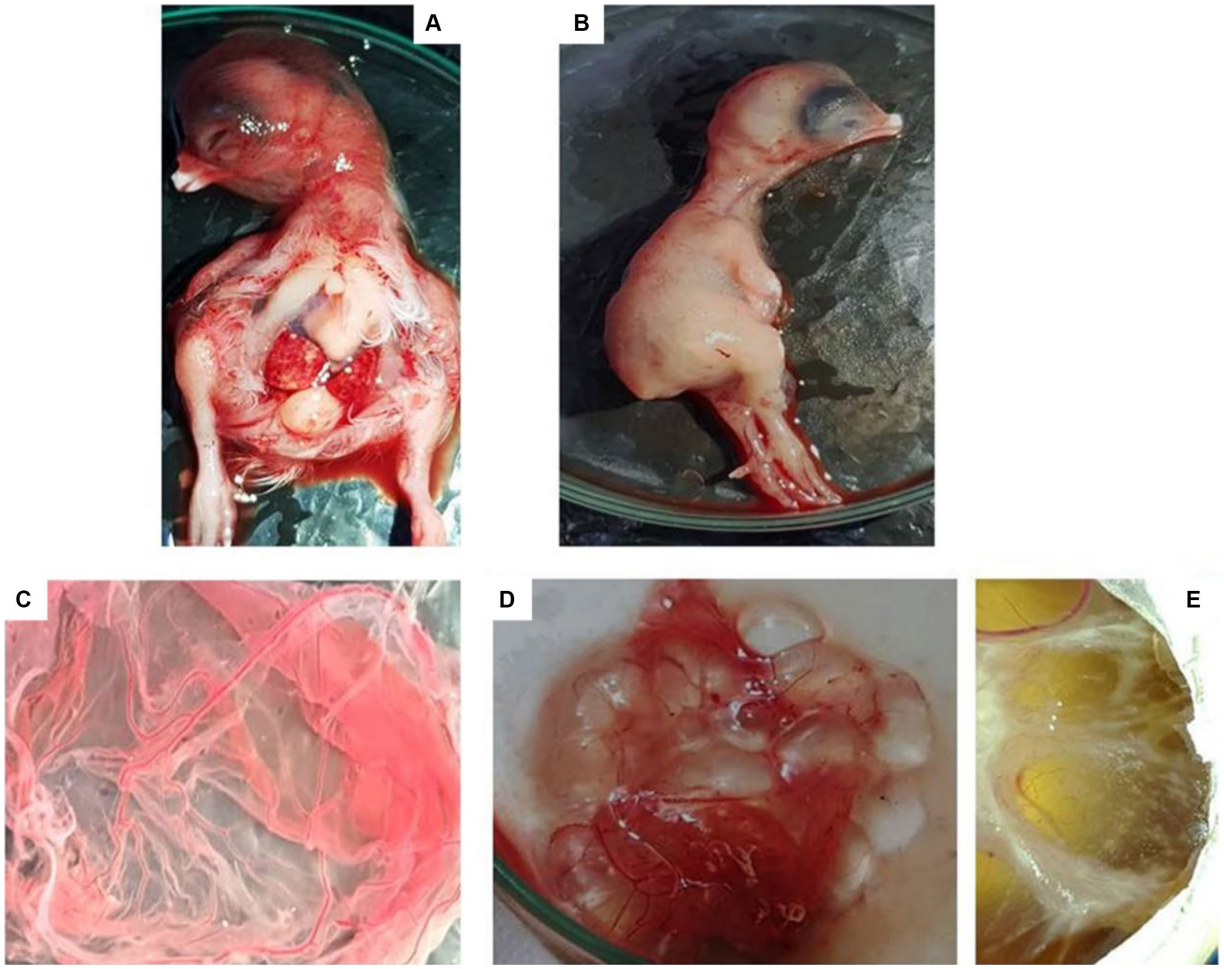
Embryo and Chorioallantoic membrane of EGEs after three passages by LSDV. Hemorrhegic and edematous embryo **(A)**, a hemorrhagic CAM with congestion and pock lesion infected by LSDV **(D–E)**. Control embryo and CAM **(B,C)**.

The isolation of LSDV resulted in the characteristic pock lesions on the Chorioallantoic Membrane (CAM) of embryonated chicken eggs (ECE). Notably, embryo mortality was evident as early as the first passage, along with the generation of pock lesions. By the third passage, observations included a hemorrhagic membrane, congestion, clotted blood within blood vessels, and pock lesions appearing as stretched white lines. These pock lesions became even more prominent 5 days after inoculation during the third passage. The dead embryos showed hemorrhagic and edematous characteristics, along with an enlarged and bloody liver, and clots of blood within the core (Figure 5). It is worth noting that all PCR-positive samples inoculated into ECE consistently produced a 192 bp PCR product after amplification of P32 gene.

### Nucleotide sequencing and phylogenetic analysis

**Figure 6:**
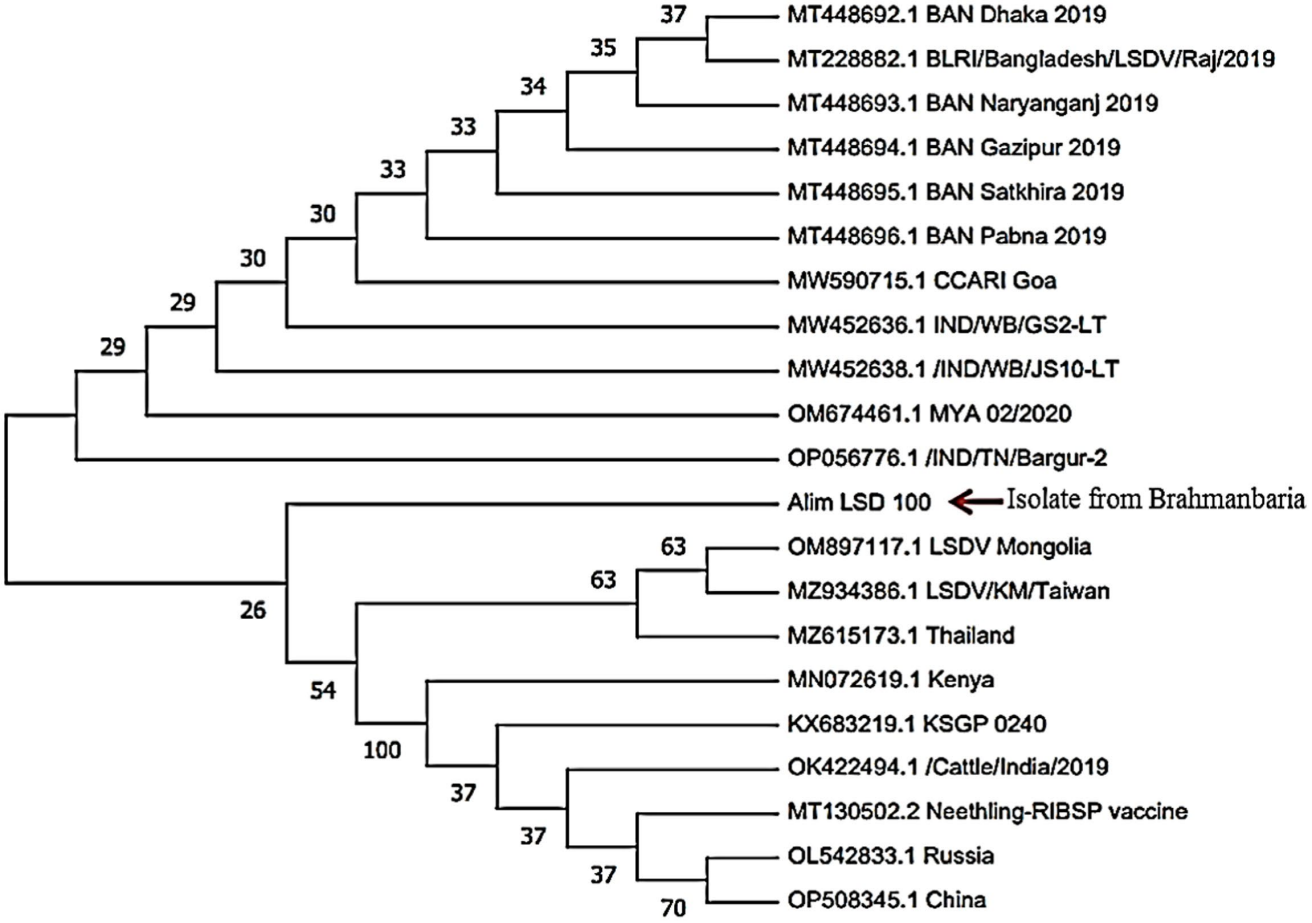
Phylogenetic analysis of the nt sequences gene of LSDV isolates of Bangladesh with the nt sequences of RPO30 gene of other countries including Bangladesh.

We successfully amplified and sequenced fragments of the RPO30 gene of LSDV. NCBI blast results revealed that the Alim_LSD_100 isolate shares a 100% nucleotide sequence similarity with all Bangladeshi LSDV isolates, as well as those from neighboring countries such as India and Myanmar. A phylogenetic analysis of LSDV was constructed based on the RPO30 gene, and it was observed that our isolate is closely related to LSDV strains from Bangladesh, India, Mongolia, and Myanmar. Importantly, it is distinctly different from LSDV strains found in Russia and China (Figure 6).

### Immunogenicity test of the selected LSD virus antigen

**Figure 7:**
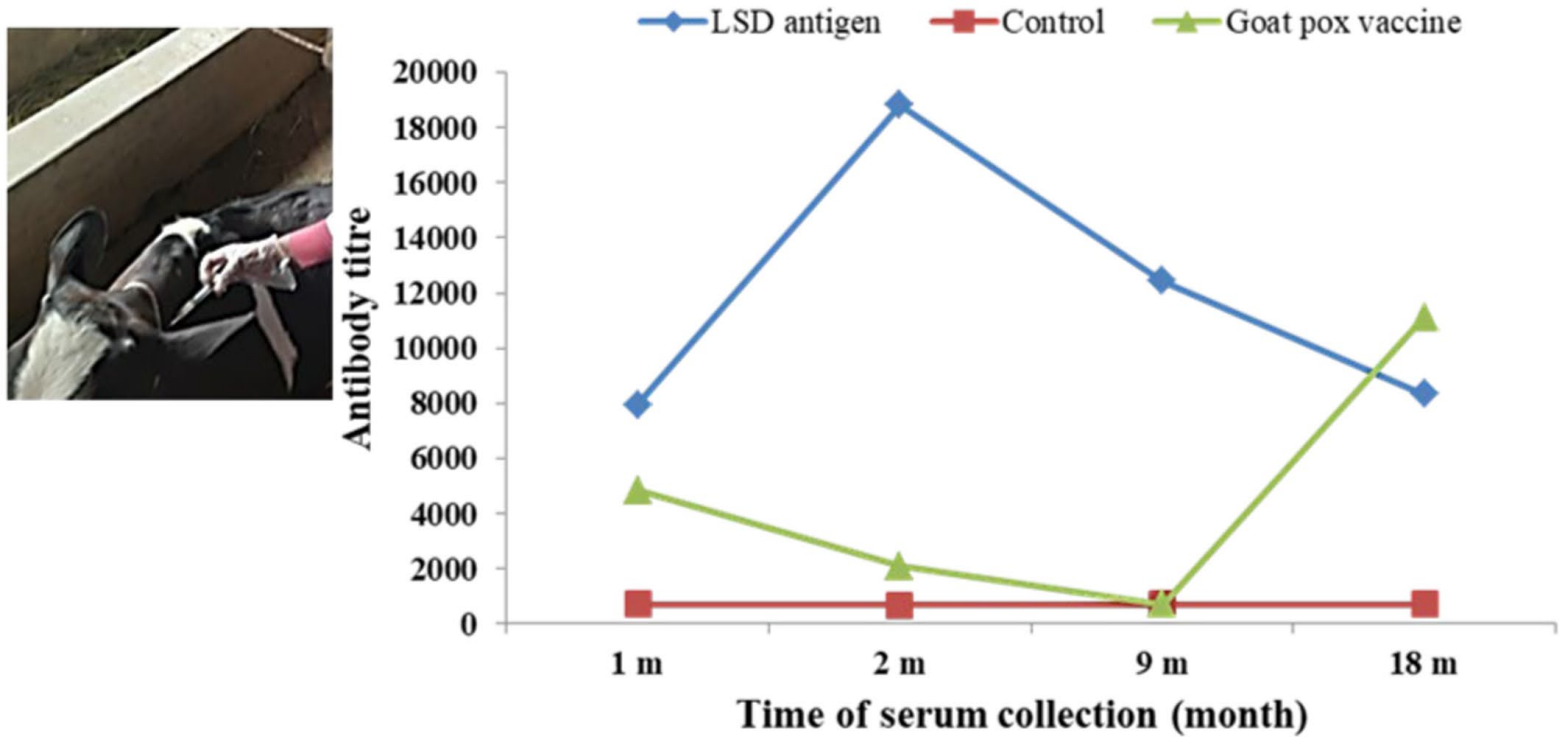
Subcutaneous immunization of cattle with the killed LSDV antigen and serum antibody titre after immunization.

In cattle immunized with LSD antigen, serum antibody titers were measured at 7929.53, 18807.99, 12425.76, and 8317.54 after 1, 2, 9, and 18 months of immunization, respectively. In contrast, in the goat pox vaccinated group, the antibody titers were 4825.79, 2070.60, 699.00, and 11099.00 after 1, 2, 9, and 18 months of immunization, respectively. For the non-immunized cattle, the antibody titer was nearly 700. The highest antibody titer was observed in the LSD-immunized group after 2 months of immunization (Figure 7).

## Discussion

We employed a novel PCR technique for the screening of nodular tissue samples to detect LSDV. Subsequently, we successfully cultivated large quantities of LSDV in chicken embryos, and we also assessed the immunogenicity of the inactivated LSDV. Our molecular evidence strongly suggests a transboundary spread of the LSD outbreak in Bangladesh during 2019–2020. Moreover, our LSDV isolates show a potential vaccine candidate to combat LSD in Bangladesh.

The iiPCR technique offers a distinct advantage in rapidly completing the denaturation, annealing, and extension steps within a capillary tube, achieving high amplification efficiency over a short time period. This feature makes it particularly suitable for swift and efficient screening of field virus infections (Song et al., 2022). In our study, iiPCR was employed to screen the nodular tissue samples for LSDV. Importantly, all iiPCR-positive samples were subsequently confirmed as PCR-positive, with a specific 192 bp band appearing for the P32 gene, a well-established marker for LSDV detection. This demonstrates the superiority of iiPCR in enabling the rapid and accurate screening of LSDV. Giasuddin et al. (2019) and Sudhakar et al. (2020) also used the P32 gene as a specific marker for detecting LSDV via PCR.

Lumpy skin disease affects the skin of cattle and water buffalo, leading to the development of nodular lesions that may rupture in advanced stages of the disease (Sanz-Bernardo et al., 2021). Microscopically, these lesions exhibit characteristic features, including acanthosis (thickening of the epidermis), ballooning degeneration of epidermal cells, and the presence of intracytoplasmic inclusion bodies (Ali et al., 2021). Notably, the presence of intracytoplasmic inclusion bodies is considered a hallmark of lumpy skin disease (Gharban et al., 2019; Neamat-Allah and Mahmoud, 2019). The skin lesions are typically demarcated from nearby healthy tissue by the presence of inflammatory infiltrates, which consist of lymphocytes and macrophage cells. Additionally, the disease leads to thickening and coagulation necrosis of subcutaneous muscle tissue (Neamat-Allah, 2015; Sanz-Bernardo et al., 2020; Parvin et al., 2022). In the present study, LSDV was successfully isolated from nodular tissues collected from the skin of naturally infected cattle through inoculation on the Chorioallantoic Membrane (CAM) of embryonated chicken eggs (ECE). During the third passage, the embryos exhibited hemorrhagic and edematous characteristics, a finding consistent with the observations made by van Rooyen et al. (1969). Characteristic pock lesions were initially observed after the first passage and became more pronounced after the third passage. This method of LSDV isolation on the CAM of ECE, along with the detection of characteristic pock lesions, has been successfully employed by several authors (House et al., 1990; Hassan et al., 1992; Tamam, 2006; El-Nahas et al., 2011; El-Tholoth and El-Kenawy, 2016). Importantly, all LSDV isolates obtained from the infected CAM were confirmed through PCR analysis. El-Nahas et al. (2011) also employed PCR to detect LSDV in infected CAM.

The RPO30 gene has consistently demonstrated its suitability as a prime candidate for the phylogenetic differentiation of field strains of LSDV, as evidenced by prior studies (Gelaye et al., 2015; Agianniotaki et al., 2017; Sudhakar et al., 2020). In our study, we utilized the partial sequence of the RPO30 gene for phylogenetic analysis. The selected LSDV isolate from Brahmanbaria district (highlighted by the red arrow in Figure 6) exhibited genetic similarities with LSDV isolates from Bangladesh and neighboring countries such as India and Myanmar. This genetic relationship highlights the critical need for regional collaboration and coordinated efforts in disease surveillance, early detection, and response mechanisms. Effective control and prevention of LSDV necessitate cross-border cooperation among neighboring countries. While the observed genetic similarities strongly suggest the possibility of transboundary transmission of LSD in Bangladesh during 2019–2020, it is crucial to note that confirmation of such spread requires the availability of whole-genome sequences, encompassing a combination of LSDV target genes.

The immune response elicited in cattle by the inactivated LSDV antigen was compared with that in goat pox-vaccinated cattle and the control group. Notably, a stronger immune response was observed in the cattle immunized with the inactivated LSDV antigen compared to the other two groups. However, it is important to highlight that LSDV infection was observed in both the goat pox-vaccinated group and the control group. The antibody titre was sharply increased in goat pox vaccinated cattle after 9 months due to the sudden outbreak of LSD 5 months after immunization. This increase in antibody titer was attributed to the recovery of the cattle from LSD infection. In contrast, there were no reports of LSDV infection recorded in the cattle immunized with the LSDV antigen.

Our study focuses on developing and evaluating an inactivated LSDV vaccine candidate. Current LSD vaccines typically use strains like Neethling or KSGP O-240 and O-180, requiring extensive passages in cell cultures and in the chorioallantoic membrane of embryonated chicken eggs (Kitching, 2003; Wallace and Viljoen, 2005). The Neethling strain vaccination can lead to adverse reactions like skin nodules and reduced milk yield (Wallace and Viljoen, 2005). Inactivated vaccines offer a shorter immunity duration but are free from adverse effects, making them favorable in disease-free, at-risk regions (Tuppurainen et al., 2021). Our study contributes to this field by exploring the potential of an inactivated LSDV vaccine candidate, offering an alternative approach with its own set of benefits and limitations.

In conclusion, this study represents a significant advancement in our understanding of LSDV in Bangladesh. We introduced an innovative PCR technique for the rapid and efficient screening of nodular tissue samples, enabling the detection of LSDV with high accuracy. Furthermore, we successfully cultivated substantial quantities of LSDV using chicken embryos. One of the most notable findings of this study is the strong molecular evidence indicating potential transboundary spread of the LSD outbreak in Bangladesh during the period 2019–2020. This highlights the interconnectedness of the region and the potential for diseases like LSDV to cross borders, emphasizing the importance of vigilance and cooperation in disease management and control efforts. Additionally, our LSDV isolates hold promise as potential vaccine candidates to combat LSD in Bangladesh. Further investigation into the effectiveness of the inactivated LSDV antigen as a vaccine candidate, as well as the development of surveillance strategies to monitor LSDV circulation in the region, is recommended.

## Data availability statement

The data presented in the study are deposited in the GenBank, accession number OP948135.

## Ethics statement

The animal studies were approved by Animal Welfare and Experimentation Ethics Committee at Bangladesh Agricultural University, Mymensingh. The studies were conducted in accordance with the local legislation and institutional requirements. Written informed consent was obtained from the owners for the participation of their animals in this study. Written informed consent was obtained from the individual(s) for the publication of any potentially identifiable images or data included in this article.

## Author contributions

MAU: Investigation, Methodology, Writing – original draft. MTH: Conceptualization, Data curation, Methodology, Supervision, Writing – original draft, Writing – review & editing. AKMAR: Data curation, Formal analysis, Writing – review & editing. MPS: Formal analysis, Writing – review & editing. MAK: Writing – review & editing. MGH: Methodology, Writing – review & editing. SC: Writing – original draft. MMR: Writing – review & editing. AKMK: Writing – review & editing. MPW: Writing – review & editing. MAI: Conceptualization, Data curation, Formal analysis, Funding acquisition, Investigation, Methodology, Supervision, Writing – review & editing.
